# Loss to Clinic and Five-Year Mortality among HIV-Infected Antiretroviral Therapy Initiators

**DOI:** 10.1371/journal.pone.0102305

**Published:** 2014-07-10

**Authors:** Jessie K. Edwards, Stephen R. Cole, Daniel Westreich, Richard Moore, Christopher Mathews, Elvin Geng, Joseph J. Eron, Michael J. Mugavero, for the CNICS Research Network

**Affiliations:** 1 Department of Epidemiology, University of North Carolina at Chapel Hill, Chapel Hill, North Carolina, United States of America; 2 School of Medicine, Johns Hopkins University, Baltimore, Maryland, United States of America; 3 School of Medicine, University of California San Diego, San Diego, California, United States of America; 4 School of Medicine, University of California San Francisco, San Francisco, California, United States of America; 5 School of Medicine, University of North Carolina at Chapel Hill, Chapel Hill, North Carolina, United States of America; 6 School of Medicine, University of Alabama at Birmingham, Birmingham, Alabama, United States of America; Infectious Disease Service, United States of America

## Abstract

Missing outcome data due to loss to follow-up occurs frequently in clinical cohort studies of HIV-infected patients. Censoring patients when they become lost can produce inaccurate results if the risk of the outcome among the censored patients differs from the risk of the outcome among patients remaining under observation. We examine whether patients who are considered lost to follow up are at increased risk of mortality compared to those who remain under observation. Patients from the US Centers for AIDS Research Network of Integrated Clinical Systems (CNICS) who newly initiated combination antiretroviral therapy between January 1, 1998 and December 31, 2009 and survived for at least one year were included in the study. Mortality information was available for all participants regardless of continued observation in the CNICS. We compare mortality between patients retained in the cohort and those lost-to-clinic, as commonly defined by a 12-month gap in care. Patients who were considered lost-to-clinic had modestly elevated mortality compared to patients who remained under observation after 5 years (risk ratio (RR): 1.2; 95% CI: 0.9, 1.5). Results were similar after redefining loss-to-clinic as 6 months (RR: 1.0; 95% CI: 0.8, 1.3) or 18 months (RR: 1.2; 95% CI: 0.8, 1.6) without a documented clinic visit. The small increase in mortality associated with becoming lost to clinic suggests that these patients were not lost to care, rather they likely transitioned to care at a facility outside the study. The modestly higher mortality among patients who were lost-to-clinic implies that when we necessarily censor these patients in studies of time-varying exposures, we are likely to incur at most a modest selection bias.

## Introduction

Missing outcome data from loss to follow-up occurs in both randomized trials and observational studies [1]. The validity of treatment (or exposure) effects is questionable in cohort studies with high amounts of loss to follow-up. In clinical cohort studies of HIV-infected patients, the evaluations of time-varying exposures, biomarkers and clinical events are typically precluded when patients cease to return for care.

In clinical HIV cohort studies, censoring is often defined by failing to return for care at a specific clinic for a pre-specified interval of time, typically ranging from 3 to 18 months. After censoring, a patient’s hazard of the outcome is assumed to be the same as the hazard for comparable patients who remain under observation [2]. If this assumption is violated, estimates of the incidence of the outcome or the effect of an exposure on the outcome may be biased. For example, if patients who are lost from the study clinic (henceforth, lost-to-clinic) do not seek care elsewhere, they may have a higher risk of mortality compared to patients who remain under observation. As an alternate example, there may be unmeasured common causes of patients becoming lost-to-clinic and the outcome, such as socioeconomic status. Here, we investigate whether patients who are considered lost-to-clinic are at increased risk of 5-year mortality compared to those who remain under observation in a multi-site clinical HIV cohort in the United States.

## Methods

### Study population

The Centers for AIDS Research Network of Integrated Clinical Systems (CNICS) was developed to maintain a comprehensive and standardized clinical data repository to support population-based HIV research in the United States [3]. The CNICS cohort includes over 27,000 HIV-positive adults engaged in clinical care from January 1, 1995 to the present at 8 CFAR sites (Case Western Reserve University; Fenway Community Health Center of Harvard University; Johns Hopkins University; University of Alabama at Birmingham; University of California, San Diego; University of California, San Francisco; University of North Carolina; and University of Washington). Institutional review boards at each site approved study procedures. Participants provided written informed consent to be included in the CNICS cohort or contributed administrative and/or clinical data with a waiver of written informed consent where approved by local institutional review board(s).

All patients attending 2 primary HIV medical care visits at study sites are included in CNICS and followed longitudinally while they remain in care at study sites. The average time between follow up visits is 3 months, however, patients can be seen more or less often depending on clinical care. CNICS is a dynamic cohort with approximately 1400 new patients enrolling and 10% of existing patients becoming lost to the cohort each year [3]. There is no CNICS-wide systematic approach to assess the disposition of patients lost-to-clinic to determine if they are truly out of HIV care or have transferred care to another medical clinic, though efforts to track patients lost-to-clinic have been undertaken at specific sites [4]. CNICS provides open access to data through its concept review process (www.uab.edu/cnics). The CNICS includes 12,590 patients who entered care at a CNICS site between January 1, 1999 and December 31, 2009 and had not previously initiated combined antiretroviral therapy. Of these 12,590 patients, we included 7635 patients who newly initiated combination antiretroviral therapy and had measured CD4 cell count and viral load between these dates. Therapy initiation was defined as the date of initiation of a regimen consisting of three or more antiretroviral agents. We did not exclude patients with documented prior mono/dual antiretroviral therapy exposure. Of the 7635 patients newly initiating therapy, 7183 (93%) survived for at least one year and were eligible for inclusion in the analysis of the relationship between becoming lost-to-clinic and death.

### Lost-to-clinic

In analyses of the CNICS and other cohorts, patients are sometimes censored after 12 months without a documented clinic visit. To determine if the risk of mortality differed between person-time that is censored and person-time that is included in such a study, we compared the risk of mortality between patients who had experienced a 12-month gap in care with the risk of mortality among patients who had not yet experienced a 12-month gap in care. After patients had a 12-month gap in care, we considered them to be lost-to-clinic throughout the remainder of the study period, as patients who are censored are usually not allowed to re-enter study. We quantify the amount of misclassified person-time due to patients returning to the clinic after a 12-month gap in care in the discussion. We also perform a sensitivity analysis in which the definition of “loss to care” was varied to 6 months and 18 months without a documented clinic visit.

### Mortality ascertainment

The outcome of interest was all-cause mortality. Each CNICS site maintains a registry of deaths among patients at that site and semiannually queries the United States Social Security Death Index and/or National Death Index to confirm reported deaths and record deaths not captured by the CNICS sites. Mortality records were linked to CNICS patients using approved Social Security Death Index and National Death Index matching criteria, including combinations of first and last names, father’s surname, social security number, and month and year of birth. Deaths among patients are captured in these queries regardless of lost-to-clinic status. Because surveillance for mortality is conducted using national registries, outcome ascertainment is uniform across the study sites.

Deaths were considered to occur “in care” unless they occurred after a 12-month gap in care. After a 12-month gap in care, deaths were classified as lost-to-clinic. Because delays in reporting to the vital status registries could give the appearance of artificially low mortality among patients lost-to-clinic (and thus not captured in clinical death registries) in the most recent years of data, we administratively censored all patients on December 31, 2010 to allow adequate time for deaths to be reported to Social Security Death Index or National Death Index and CNICS.

### Statistical methods

All patients were in care at a study clinic at ART initiation by definition and were eligible to become lost to clinic after surviving for one year. Patients were followed from one year after ART initiation until death, December 31, 2010, or 6 years after ART initiation, for a maximum follow-up time of 5 years. Patients at two study sites were censored on December 31, 2009 due to incomplete visit data in 2010.

We compared cumulative incidence of mortality between patients remaining in care at a study clinic and those who were lost-to-clinic using risk ratios and risk differences [5]. To estimate the effect of becoming lost-to-clinic on mortality in the presence of time-varying confounding, we used marginal structural models to estimate risk ratios and risk differences that were standardized to the total study sample. Standardized risk ratios and risk differences were estimated using stabilized inverse probability weights [6]–[8] constructed from a pooled logistic regression model for becoming lost-to-clinic, with time coarsened to the month. Weights were estimated using time-fixed and time-varying covariates. Time-fixed covariates were measured at baseline one year after therapy initiation and included sex, age, race, ethnicity, AIDS status, history of single or two-drug antiretroviral therapy, sexual orientation, injection drug use, CD4 cell count and viral load, and calendar date of ART initiation. Time-varying factors were updated monthly and included the patient’s AIDS status, CD4 cell count, and viral load (averaged over the previous month). Continuous variables (age, CD4 cell count, viral load, and calendar date of ART initiation) were modeled using restricted quadratic splines with 4 knots placed at the 5^th^, 35^th^, 65^rd^, and 95^th^ percentiles [9]. Because lost-to-clinic was defined after a 12-month absence, time-varying covariates were lagged 12 months in the models for becoming lost-to-clinic used to construct the weights. The inverse probability weights had a mean of 1.00 (standard deviation: 0.34) and ranged from 0.24 to 9.25. The standardized mortality estimates were calculated using the complements of the Kaplan-Meier survival curves in the weighted data [10]. Ninety-five percent confidence intervals for risk ratios and risk differences were calculated using standard errors estimated by the standard deviation of the effect measures in 200 nonparametric bootstrap [11] samples with replacement of the original study sample size. Statistical analyses were performed using SAS version 9.3 (SAS Institute, Inc., Cary, NC).

## Results

Table 1 describes the characteristics of the 7183 patients at study entry one year after ART initiation and during 25,581 person-years of follow-up. At the start of follow up, 18% of patients were female, 39% were African-American, 94% were mono/dual antiretroviral-therapy naïve, and 32% had a prior AIDS diagnosis. The median calendar year was 2004 (IQR: 2001, 2007), and the median age was 39 years (IQR: 33, 35). At baseline, the median CD4 cell count was 326 (IQR: 50, 759), and 73% had a suppressed viral load, defined as under 500 copies/mL. Among the unsuppressed, the median log_10_ viral load was 4.4 (IQR: 3.6, 5.0).

**Table 1:** Demographics and clinical characteristics at study entry one year after ART initiation and over 25,581 person-years of follow-up among 7183 patients who initiated antiretroviral therapy between January 1, 1998 and December 31, 2009 and survived for at least one year at 8 US clinical sites, followed for death up to 5 years.

Characteristics	Study entry *N* = 7183 patients		Retained in care *n = *17897 person-years	Lost to clinic *n = *7684 person-years
	*n*	%	%	%
Male sex	5817	82	81	81
Black race	2830	39	40	39
Hispanic ethnicity	888	12	12	12
Injection drug user	1156	16	16	18
MSM	4092	57	56	55
Prior ARV use	460	6	7	8
AIDS	2279	32	38	– a
CD4 cell count				
<250	2605	36	23	– a
250–500	2726	38	38	– a
>500	1852	26	39	– a
Suppressed viral load^b^	1909	73	79	– a

ARV, antiretroviral; IQR, interquartile range; MSM, men who have sex with men; ART, antiretroviral therapy; AIDS, acquired immunodeficiency syndrome; VL, viral load.

a Unavailable.

b <500 copies/ML.

**Figure 1:**
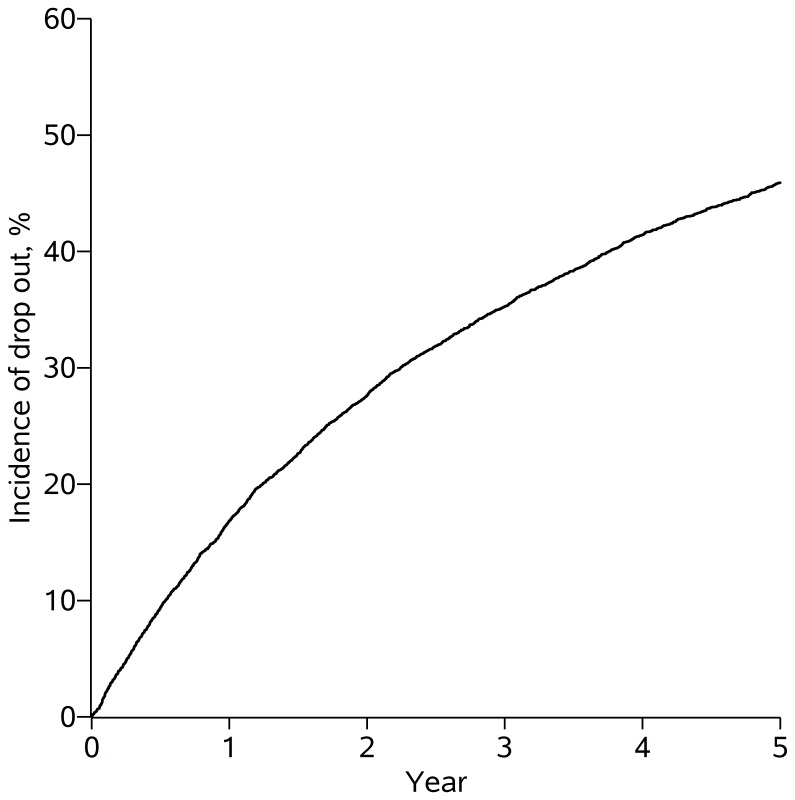
Cumulative incidence of loss to clinic. Loss to clinic was defined as a 12-month absence from CNICS clinics. The figure presents loss to clinic among 7183 patients who initiated antiretroviral therapy between January 1, 1998 and December 31, 2009 at 8 US clinical sites and survived for at least one year, followed up for 5 years.

Over the 25,581 person-years of follow-up, 3080 of 7183 patients experienced a 12-month gap in care, rendering them lost-to-clinic by our definition. The cumulative incidence of lost-to-clinic was 46% at 5 years (Figure 1). During the 17,897 person-years contributed by patients in care at CNICS sites prior to their first 12-month gap in care, the median CD4 cell count was 427 (IQR: 262, 617) and patients’ viral loads were suppressed for 79% of the in-care person-years. The median log_10_ viral load during the person-years in which patients’ viral loads were unsuppressed was 4.4 (IQR: 3.5, 5.0). By definition, CD4 cell count and viral load measurements were not available during the 7684 person-years lost to clinic, during which patients were not evaluated at CNICS sites, but remained alive according to national vital status indices.

**Figure 2:**
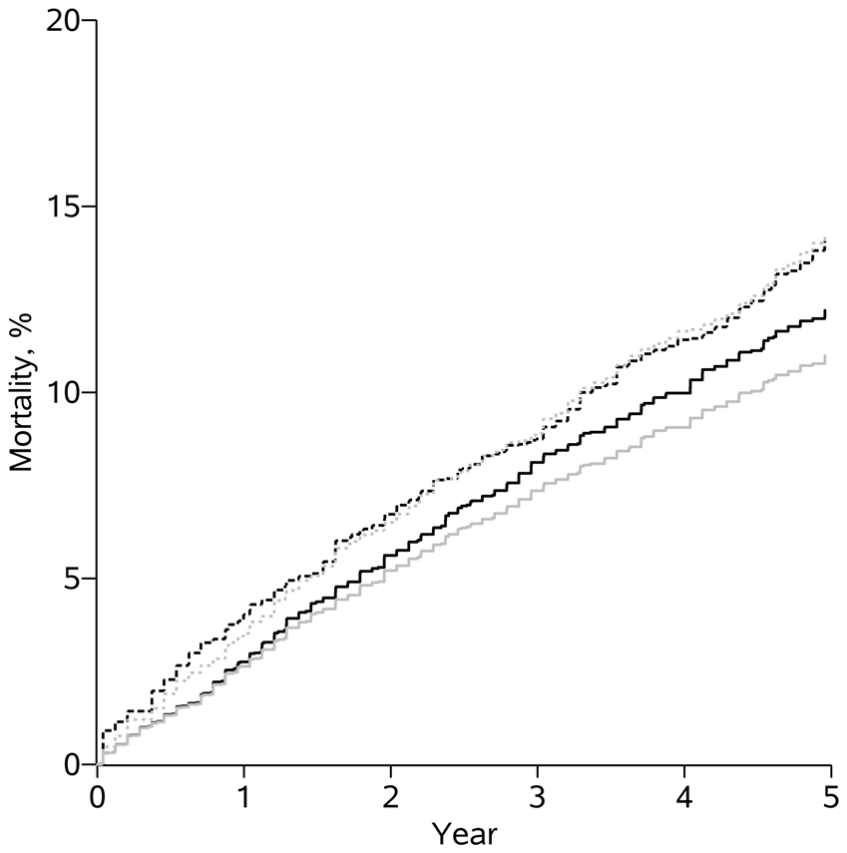
Cumulative mortality for patients in care and lost to clinic. Crude (grey) and standardized (black) survival curves compare mortality between patients continuously retained in care at CNICS sites (solid lines) and patients lost to clinic (dotted lines) among 7183 patients who initiated antiretroviral therapy between January 1, 1998 and December 31, 2009 and survived for at least one year at 8 US clinical sites, followed for death up for 5 years.

Over the 5 years of follow-up, 439 deaths occurred during the 17,987 person-years contributed by patients in care at CNICS sites. During the same time-period, among the 7684 person-years contributed by patients lost-to-clinic, 229 deaths occurred. Table 2 provides the crude and standardized 5-year cumulative mortality risk, risk ratios, and risk differences for patients retained in care at CNICS sites and those lost. The crude 5-year risk difference was 3.26% (95% CI: 0.99, 5.54) and risk ratio was 1.29 (95% CI: 1.09, 1.53) for patients lost-to-clinic relative to those remaining in care. Because few measured variables predicted which patients became lost-to-clinic, the estimated risk difference and risk ratio comparing mortality for patients lost-to-clinic and those remaining in care were relatively similar after standardization by baseline and time-varying factors; the standardized 5-year risk difference was 2.22% (95% CI: −1.38, 5.86) and risk ratio was 1.18 (95% CI: 0.92, 1.50). Figure 2 provides the crude and standardized cumulative mortality curves for those retained continuously in care at CNICS sites and those lost-to-clinic.

**Table 2:** Five-year risk ratios and risk differences comparing mortality between patients continuously retained in care at CNICS sites and patients lost to clinic among 7183 patients who initiated antiretroviral therapy between January 1, 1998 and December 31, 2009 and survived for at least one year at 8 US clinical sites, followed for death up to 5 years.

		Deaths	Person-years	Mortality^a^	RR (95% CI^b^)	RD (95% CI^b^)
Crude	In care	439	17896.76	10.99	1	0
	Lost to clinic	229	7684.28	14.21	1.29 (1.09, 1.53)	3.26 (0.99, 5.54)
Standardized^c^	In care	487.2	18033.17	12.20	1	0
	Lost to clinic	213.8	7479.18	14.06	1.18 (0.92, 1.50)	2.22 (–1.38, 5.86 )

CI, Confidence interval; RR, risk ratio; RD, risk difference.

a Cumulative mortality risk was calculated as the complement of the Kaplan-Meier survival curve at 5 and 10 years.

b Confidence intervals based on 200 nonparametric bootstrap resamples.

c For sex, age, race, ethnicity, AIDS status at baseline, antiretroviral-therapy-naive at baseline, sexual orientation, injection drug use at baseline, CD4 cell count, viral load at baseline, and calendar date of ART initiation, and time-varying CD4 cell count, viral load, and AIDS status.

Results were similar under alternate definitions of loss to clinic. When loss to clinic was defined as 6 months without a clinic visit, the standardized 5-year risk ratio was 1.03 (95% CI: 0.84, 1.26) and when loss to clinic was defined as 18 months without a clinic visit, the standardized 5-year risk ratio was 1.16 (95% CI: 0.84, 1.60).

## Discussion

In the CNICS cohort, the 5-year risk of becoming lost-to-clinic, defined as a 12-month gap in care after surviving for 1 year from combination ART initiation, was 46%. The large number of patients with a 12-month gap in care by 5 years is a concern for both clinicians and researchers. For clinicians, a patient becoming lost-to-clinic for 12 months or longer may signal lack of access to the health system, non-adherence to ART, and, subsequently, greater plasma viremia conferring poor prognosis and higher probability of transmission [12], [13]. For researchers, patients who are lost-to-clinic may not provide necessary outcome (and other) data to include in studies, meaning that outcome data for patients in care at study sites must represent the missing outcome data for those who are lost-to-clinic. If patients lost-to-clinic have different experiences than patients in care at CNICS sites, such studies might produce inaccurate results [14], [15].

However, in the CNICS cohort, patients who were lost-to-clinic for 12 or more months had only 1.2 times the risk of mortality of patients retained in care. The elevation in mortality among those lost to clinic was modest compared to the effect seen in cohorts in developing countries, where patients lost to clinic may have up to 10 times the risk of mortality of patients retained in care [16]–[19].

The difference in the effect of becoming lost to clinic in the CNICS cohort and in developing countries could stem from heterogeneity in both the reasons for becoming lost-to-clinic and the experiences of patients after becoming lost to clinic. In both settings, patients who are lost to a particular clinic may or may not have disengaged from care entirely. For example, patients who become lost to one clinic may have transferred to a different health services provider and treatment center [20]. Other patients may have become lost not only at the clinic of record but altogether, resulting in cessation of all treatments, increased risk for opportunistic infections, and earlier mortality [21], [22]. While we expect that our results are generalizable to clinical cohort studies of patients with HIV in the United States, the modest increase in mortality among patients lost to clinic in the CNICS may not be generalizable to settings in which most or all patients who are lost to clinic, in fact, become lost to care altogether.

In settings without comprehensive death registries, death itself can be a reason that a patient becomes lost-to-clinic [23]. In this situation, common in resource limited settings, we would expect to observe a stronger association between those lost-to-clinic and death than in settings, such as the CNICS, in which comprehensive death registries are available. On the other hand, in settings without a comprehensive death registry, deaths occurring after patients become lost to clinic could be underreported if systematic tracking efforts fail to recover all deaths among patients who are lost [24].

In the CNICS cohort, reasons for absence from care and the experiences of patients during gaps in care are unknown. The modest increase in mortality among patients lost to clinic suggests a large fraction of these patients are more likely to have transferred to another health provider or to have re-engaged in care at a CNICS site after a 12-month gap than to have become disengaged from the health system entirely. In our analysis, we consider patients to be lost to clinic after the first 12-month gap in clinic visits in which the patient had a lab test (i.e., CD4 cell count or viral load assessment) or initiated ART. Some patients returned to care at a CNICS site later in the study period, but after patients were classified as lost to clinic, we considered them to be lost throughout the remainder of follow up, as this is how they would typically be handled analytically by censoring. Person-time of patients who were lost but then returned to care is misclassified. In the CNICS cohort, 39% (*n* = 1110) of the 2828 patients but only 10% of the person-years who became lost to clinic by our definition returned to care at a site in the CNICS during the 5-year study period. While we would be interested in comparing mortality between patients who returned to care and patients who remained lost to the clinic, we could not account for confounding in such a comparison because predictors of return to care were not measured after patients left care at a CNICS site. Notably, altering the definition of becoming lost to clinic had only a modest impact on the 5-year risk ratio.

A number of approaches have been developed to measure retention in HIV care, with no clear gold standard established [25]. Most prior studies evaluating the impact of HIV care retention on health outcomes have measured retention over relatively short observation periods, typically 1–2 years [26]–[28]. Here, we measure retention using 12-month gap by monitoring participants for this event over a considerably longer 5-year observation period. Our finding that almost half of CNICS participants had a 12-month gap within 5 years of starting ART is striking. Gaps of this length may become more common as patients on stable therapy with suppressed viral load and high CD4 cell counts may be seen by primary providers outside the HIV clinic setting and only return to more specialized care at greater intervals or when HIV specific treatment decisions are needed. The reason for large gaps in cohort clinic participation can be examined in those patients who reconnect to care. The observation that 39% of those experiencing a 12-month gap re-connect to care within the 5-year study period suggests that future studies of this group may be informative [29].

The magnitude of the associations presented here reflects both the effect of lost-to-clinic on mortality and the strength of the unmeasured common causes of becoming lost-to-clinic and death. While we included measured predictors in the estimation of the standardizing weights, it is likely that unmeasured variables also acted as predictors of both becoming lost-to-clinic and mortality. For example, we did not account for income; if patients with lower income were more likely to become lost-to-clinic and had higher risk of mortality, then our estimates of the effect of loss-to-care on mortality might have an upward bias.

Censoring patients who are lost-to-clinic is often necessary when loss-to-clinic precludes observation of time-varying covariates or outcomes. The higher mortality among patients with a 12-month gap in care means that censoring patients who are lost-to-clinic for 12 or more months may result in an underestimate of absolute mortality in the population under study [16], [17]. In addition, censoring patients who are lost-to-clinic may induce selection bias in studies of treatment or exposure effects. For selection bias to occur there must be: 1) selection (i.e. some patients must be lost-to-clinic); 2) loss-to-clinic associated with exposure; and 3) loss-to-clinic associated with the outcome [14]. While the first two conditions above can usually be assessed in observed data, the present work provides rare insight into the third condition, which is typically not estimable in observed data. The modestly higher mortality among patients who were lost-to-clinic implies that when we necessarily censor these patients in studies of mortality or other related outcomes, we are likely to incur at most a modest bias in the risk or estimates of exposure effects on these outcomes. However, if the association between loss-to-clinic and mortality is due solely to the direct effect of loss-to-clinic on mortality, the elevation in mortality among patients lost to clinic means only that total effect of an exposure will differ from the effect of that exposure estimated when patients lost to clinic are censored.

Beyond the methodological and analytic implications of our findings for evaluation and inference from HIV clinical cohort studies, we make novel observations of clinical and public health importance. Our finding that an incident 12-month gap occurring up to 5 years following ART start is associated with an elevated mortality risk is germane to the contemporary HIV clinical, policy and public health agenda [26]. In the context of HIV management as a chronic disease and with increasing attention to the HIV care continuum, evaluating the dynamic nature of retention in care over the longer term is of the utmost importance.

Censoring patients when they become lost-to-clinic is often necessary in HIV or other clinical cohort studies. Patients becoming lost-to-clinic for 12 months or longer in the CNICS cohort had modestly elevated risk of mortality when compared to patients retained in care at CNICS sites. The small increase in mortality associated with becoming lost to clinic suggests that these patients were not lost to care, rather they likely transitioned to care at a facility outside the study. Because the increase in mortality for patients lost-to-clinic was small, censoring these patients is unlikely to induce substantial selection bias in studies of mortality.
